# Impaired Cortico-Thalamo-Cerebellar Integration Across Schizophrenia, Bipolar II, and Attention Deficit Hyperactivity Disorder Patients Suggests Potential Neural Signatures for Psychiatric Illness

**DOI:** 10.21203/rs.3.rs-4145883/v1

**Published:** 2024-03-29

**Authors:** Stacy N. Hudgins, Adrian Curtin, Joseph Tracy, Hasan Ayaz

**Affiliations:** Drexel University; Drexel University; Thomas Jefferson University; Drexel University

**Keywords:** Cognitive Dysmetria, Cerebellum, Thalamus, Functional Connectivity, Functional Magnetic Resonance Imaging (fMRI)

## Abstract

Understanding aberrant functional changes between brain regions has shown promise for characterizing and differentiating the symptoms associated with progressive psychiatric disorders. The functional integration between the thalamus and cerebellum significantly influences learning and memory in cognition. Observed in schizophrenic patients, dysfunction within the corticalthalamocerebellar (CTC) circuitry is linked to challenges in prioritizing, processing, coordinating, and responding to information. This study explored whether abnormal CTC functional network connectivity patterns are present across schizophrenia (SCHZ) patients, bipolar II disorder (BIPOL) patients, and ADHD patients by examining both task- and task-free conditions compared to healthy volunteers (HC). Leveraging fMRI data from 135 participants (39 HC, 27 SCHZ patients, 38 BIPOL patients, and 31 ADHD patients), we analyzed functional network connectivity (FNC) patterns across 115 cortical, thalamic, subcortical, and cerebellar regions of interest (ROIs). Guiding our investigation: First, do the brain regions of the CTC circuit exhibit distinct abnormal patterns at rest in SCHZ, ADHD, and BIPOL? Second, do working memory tasks in these patients engage common regions of the circuit in similar or unique patterns? Consistent with previous findings, our observations revealed FNC patterns constrained in the cerebellar, thalamic, striatal, hippocampal, medial prefrontal and insular cortices across all three psychiatric cohorts when compared to controls in both task and task-free conditions. Post hoc analysis suggested a predominance in schizophrenia and ADHD patients during rest, while the task condition demonstrated effects across all three disorders. Factor-by-covariance GLM MANOVA further specified regions associated with clinical symptoms and trait assessments. Our study provides evidence suggesting that dysfunctional CTC circuitry in both task-free and task-free conditions may be an important broader neural signature of psychiatric illness.

## 1. Introduction

Psychiatric research strives to characterize physiological and psychological features of illness to treat the underlying etiologies of cognitive impairments. Understanding functional impairments in the brain’s ability to adaptively engage with changing contexts has emerged as a foundation to our understanding of substrates of psychopathology (Menon, 2020). Thalamic functional integration with cerebellum plays a pivotal role in the intricate cognitive dimensions of learning and memory. Normally functional connectivity (FC) of the cortical-thalamo-cerebellar (CTC) circuitry connects thalamic regions via cortical loops through specific cerebellar regions (crus 1–2) and is prominent in working memory, adaptive control, self-reflection and language-related cognitive processes (Habas, 2021; Schmahmann et al., 2019; Stoodley et al., 2016; Stoodley & Schmahmann, 2018).

For several decades, dysfunctional CTC circuitry was implicated in patients with schizophrenia, uncovering the cerebellar role in impaired nonmotor cognitive function (Phillips et al., 2015). Whole-brain MRI studies in these patients revealed common structural and functional abnormalities within the CTC circuitry involving the prefrontal cortex, medial temporal cortex, thalamus, striatum, and cerebellum (Andreasen & Pierson, 2008; Bernard & Mittal, 2015; Collin et al., 2011; Habas, 2021; Hwang et al., 2021; Kasparek et al., 2012; Matsuo et al., 2013; Pinheiro et al., 2021; Ramsay, 2019; Wheeler & Voineskos, 2014).

Recent evidence suggests that these changes may extend beyond schizophrenia, with commonalities found in patients with ADHD and BIPOL (Cui et al., 2022; Gong et al., 2020; Hwang et al., 2021; Lupo et al., 2019; Marin et al., 2020; Phillips et al., 2015; Pinheiro et al., 2021; Ramsay, 2019; Schmahmann et al., 2019; Shinn et al., 2017; Sorella et al., 2019; Wolff & Vann, 2019). However, psychiatric implications of CTC circuitry variation have not been thoroughly investigated, nor alternations during cognitive control processes engaging the salience, frontoparietal, and default-mode networks (DMN) (Greicius et al., 2003; Seeley et al., 2007).

The current study addresses this gap by examining functional patterns and behavioral features of cognitive dysmetria in the context of SCHZ, ADHD, and BIPOL disorders. Varied functional neuroimaging responses may be attributed to the semiological degree in the underlying psychiatric spectrum or to the experimental conditions (task-free or task-based).

## 2. Methods

### 2.1. Participants

Subjects were selected from an open access neuroimaging dataset from the UCLA Consortium for Neuropsychiatric Phenomics (CNP), 135 adult male and female subjects between the ages of 21 and 50 comprised of 39 healthy volunteers (HC), 27 Schizophrenic (SCHZ), 38 Bipolar (BIPOL), and 31 attention deficit hyperactive disorder (ADHD) subjects (Gorgolewski et al., 2017; Poldrack et al., 2016). Table 1 summarizes the demographic and clinical assessment group descriptive statistics for the participants included in this study. No significant differences found in age or sex distribution within or across diagnosis groups. Diagnoses were rendered following the DSM-IV TR criteria (American Psychiatric Association, 2000) through the Structured Clinical Interview for DSM-IV and SCID-I (American Psychiatric Association, 2000) and complemented by the Adult ADHD Interview to better characterize a lifetime history of ADHD in adults (Kaufman et al., 2000). In this study, 5 different behavioral assessments were compiled for symptoms and traits for each subject (further discussion can be found in the Supplemental section).

### 2.2. Study Design and Procedures

Subjects attended two order-counterbalanced experimental sessions, one resting-state session and one cognitive session. During the resting-state session, subjects were relaxed with their eyes open while a 304-second fMRI scan was acquired. During the cognitive session, two scanning sessions were performed to assess declarative memory encoding and retrieval using paired-association memory (PAM) tasks (Poldrack et al., 2016). The encoding session (PEncode) consisted of 40 ‘memory’ trials and 24 ‘control’ trials (64 total), lasting 8.07 minutes per subject. During each memory trial, two words were presented for 1s adjacent to each other. Then, line drawings of two objects matching the words appeared above the words and were presented together for 3 more seconds (4s total). One object was traced in black and white, and the other was traced using a single color. The control trials consisted of scrambled stimuli pairs appearing for 2s, one black and white and one colored stimulus. After each trial, the subject indicated which side of the screen contained the colored object and to remember the objects and their relationships.

The retrieval session consisted of 40 correct trials (PCorrect), 40 incorrect trials (PIncorrect), and 24 control trials (total 104) and lasted 8.93 minutes per subject. For each retrieval memory trial, two adjacent objects were presented for 3s, after which the subjects were asked to rate their recall confidence. In the 40 correct trials, items were shown paired as they had been during the encoding session. During the 40 incorrect trials, items were shown paired differently than they were during the encoding session. The control trials consisted of scrambled stimuli pairs appearing for 2s.

### 2.3. Acquisition and processing

fMRI data were acquired on 3T Siemens Trio scanners using a T2-weighted echoplanar imaging (EPI) sequence following: 4 mm slice thickness, 34 slices, 2 s TR, 30 ms TE, 90° flip angle, 64×64 matrix, 192 mm FOV, and oblique slice orientation. The encoding session comprised 242 TRs, 104 (42.9%) of which were considered active tasks. The retrieval session comprised 268 TRs, 156 (58.2%) of which were considered to be active tasks. Subject scans containing known “ghost” artifacts were excluded. Further descriptions of the image technical validation and quality control methods used are provided by (Poldrack et al., 2016).

Functional images were realigned, unwarped, and slice-time corrected. Three functional-based parcellation schemes were used to define 45 ROIs within the cerebellum (Ren et al., 2019), 16 ROIs within the cerebral cortex (Schaefer et al., 2018), and 54 ROIs within the subcortex (Tian et al., 2020) (further discussion can be found in the supplemental section). 115 ROIs (cortical = PFC, insular, and angular; cerebellum = crus I-II, lobules VI, and VIIb; subcortical = hippocampus, amygdala, thalamus, and basal ganglia) were selected as seeds for between-source contrasts for both resting-state and generalized psychophysiological index (gPPI) (McLaren et al., 2012) analyses based upon reported regions associated with the CTC circuit and PAM tasks. Functional data was segmented, normalized and coregistered to MNI space, as well as motion-corrected. Accepted data were then bandpass filtered and linear detrended with no spiking. Connectivity maps were generated for each subject in both task and task-free conditions. More detailed methods are provided in the supplemental material.

### 2.4. Data analysis

Second-level within-group and within-subject analyses of covariance for FC differences across the subject groups (main contrast analysis: HCs vs. SCHZ; HCs vs. BIPOL; HCs vs. ADHD patients) were conducted via 4×3 mixed ANOVA. In both task-free and task conditions, hierarchical clustering of all ROI pairs examined for within- and between-intrinsic network connectivity used ROI-to-ROI anatomical proximity and functional similarity metrics to determine functional network connectivity sets (FNC; further details found in the supplemental material) that survived multivariate parametric GLM testing and familywise error control with the connection threshold set at p < 0.001 uncorrected and the cluster threshold set at p < 0.05 false discovery rate (FDR) (Jafri et al., 2008). Within each surviving FNC, set significant seed-target ROI pairs were identified where the connection threshold was set at p < 0.05 p-FDR correction.

## 3. Results

### 3.1. Resting-state FNC Patterns

**Figure 1:**
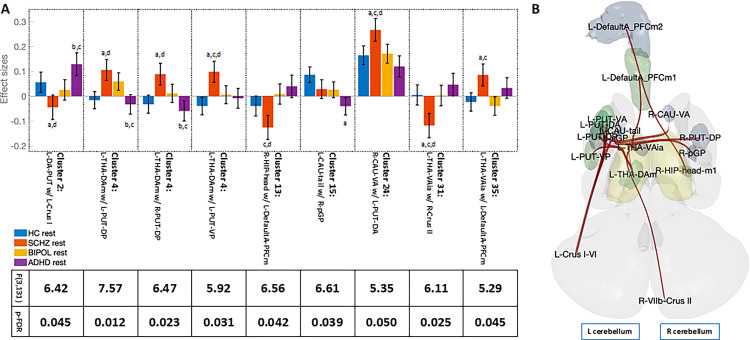
Resting-state functional network connectivity (FNC) analysis in patients with schizophrenia and bipolar and attention deficit hyperactivity disorders compared to healthy controls. Cluster-based inference connections and cluster thresholds for 231 clusters. 4×3 mixed ANOVA interaction, between-subject contrast schizophrenia patients compared to healthy controls (SCHZ>HC), bipolar (BIPOL>HC), and ADHD (ADHD>HC) patients. A: Bar graphs represent the individual effect sizes within each cluster with the corresponding F-statistic and p-FDR value shown below. Post hoc Bonferroni multiple comparisons assumed equal variances (p < 0.05): a) versus HCs, b) versus SCHZ, c) versus BIPOL, and d) ADHD. B: Seed-based ROI-to-ROI representation for significant ROIs found within a cluster (seed = regional parcel and ROI-to-ROI connection represents the F-statistic).

7 FNC sets survived multivariate parametric testing out of 231 clusters by comparing resting-state group differences between SCHZ, BIPOL, and ADHD patients and HCs, (F[3, 131] > 5.82, p-FDR > 0.0009). Within significant FNC sets, hypoconnected regions were observed between the prefrontal cortex, cerebellum, thalamus, hippocampus, and basal ganglia. Six of the seven clusters had only one significant paired connection each (Fig. 1A): between the left anterior putamen and left cerebellar crus I (p-FDR = 0.045); between the right hippocampal head and left medial prefrontal cortex (p-FDR = 0.042); between the left caudate tail and right posterior globus pallidus (p-FDR = 0.039); between the right anterior caudate and left anterior putamen (p-FDR = 0.050); between the left ventral anterior thalamus and right cerebellar crus II (p-FDR = 0.025); and between the left ventral anterior thalamus and left medial prefrontal cortex (p-FDR = 0.045). One cluster contained three significant pairs, all three with the left dorsal anterior medial thalamus and bilateral dorsal posterior putamen, as well as the left ventral posterior putamen (p-FDR < 0.031).

A post hoc analysis of multiple comparisons determined whether one or more patient groups predominated individual ROI pairs compared to the HCs, which drove the overall cluster significance (Fig. 1A: a versus HC, b versus SCHZ, c versus BIPOL, and d versus ADHD at p < 0.05 Bonferroni-corrected). In any of the comparisons, post hoc analysis revealed that BIPOL was not significantly different from any other group, whereas either SCHZ or ADHD predominated the post hoc analysis.

### 3.2 gPPI Task FNC Patterns - Paired-Association Memory (PAM)

**Figure 2:**
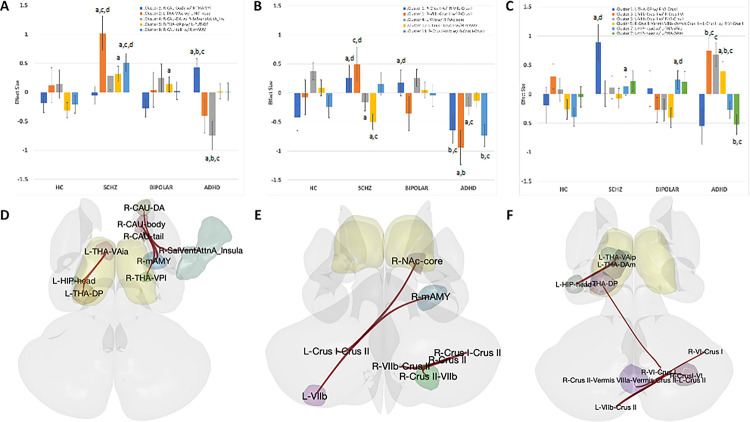
Paired-association memory induction of significant ROI-to-ROI pairs within survived functional network connectivity (FNC) sets for generalized psychophysiological indexes (gPPI) compared to healthy controls. A&D) PAM encoding. B&E) The correct match in PAM retrieval. C&F) PAM retrieval incorrectly matching. Cluster-based inference with cluster and connection thresholds at p<0.05 was FDR-corrected (clusters: 185, 166, and 204). 4×3 mixed ANOVA interaction, between-subject contrast schizophrenia compared to healthy controls (SCHZ>HC), bipolar (BIPOL>HC), and ADHD (ADHD>HC) and within-subject contrast (A&D: PAMENC > Control, B&E: PAMRET-Correctly, C&F: PAMRET-Incorrectly). Bar graphs represent individual effect sizes within each cluster with corresponding F-statistics and p-FDR values. The data in the bar graphs represent post hoc Bonferroni multiple comparisons assuming equal variances, p < 0.05 significance: a) versus HCs, b) versus SCHZ, c) versus BIPOL, and d) ADHD. D-F: Seed-based ROI-to-ROI representation of significant ROIs found within clusters (seed = regional parcel and ROI-to-ROI connection represents the F-statistic).

Using the task-modulated effective connectivity gPPI measure between ROIs, group differences between SCHZ patients, BIPOL patients, and ADHD patients from HCs in the PAM encoding (PEncode), PAM retrieval “correct” (PCorrect), and PAM retrieval “incorrect” (PIncorrect) task conditions were compared and generated 185, 166, and 204 clusters, respectively. Three to four FNC sets were observed (F[3, 131] > 5.54, p-FDR < 0.05). As part of the surviving FNC sets in the PEncode between-conditions contrast (Fig. 2A&D), significant paired ROIs arose from five sources—the right anterior caudate, body, and tail as well as the bilateral ventral anterior thalamus. In the PCorrect between-conditions contrast (Fig. 2B&E), significant cerebellar paired ROIs arose from right crus II, lobule VIIb and left crus I. For the PIncorrect condition (Fig. 2C&F), significant paired ROIs arose from the left hippocampal head, left posterior thalamus, and overlapping cerebellar vermis with bilateral crus II, left lobule VIIb, and left crus II.

Again, a post hoc analysis of multiple comparisons was conducted to discern predominant ROI pairs compared to those of HCs (Fig. 2A–C: a versus HCs, b versus SCHZ, c versus BIPOL, and d versus ADHD at p < 0.05 Bonferroni-corrected). Between-group analysis of the PEncode condition revealed significance for SCHZ, as well as for ADHD patients compared to all the other groups. In both the SCHZ and BIPOL groups, compared to the HCs, one ROI pair was observed. Between-group significance in the PCorrect condition was observed in three ROI pairs in the SCHZ group (cerebellum and amygdala), four ROI pairs in the ADHD group (cerebellum and nucleus accumbens) and one cerebellar pair in the BIPOL group. Analysis of the PIncorrect condition revealed two significant ROI pairs in the SCHZ group, one significant ROI pair in the BIPOL group, and four significant ROI pairs in the ADHD group involving the cerebellum, hippocampal head, and dorsal thalamus.

### 3.3. Significant ROIs from FNC sets compared to those from symptom and trait assessments.

Using between-subject effects, we tested for interactions between significant pairwise ROIs (covariates) and symptom/trait assessments (factors). We conducted GLM MANOVA tests of between-subjects effects where previously determined ROIs from FNC clusters covaried (Table 2: F-statistic (p value) shown for p < 0.05 significance; Supplement Table 1 provides trending for p < 0.10). We used a full-factorial design in the task-free condition and factor-by-covariate design in the task conditions.

#### 3.3.1. Task-free conditions (174)

We observed significant interactions between the HSCL subscales (the obsessive-compulsive, depression, and global severity subscales) and the pairwise ROI functional relationships between the left caudate tail and right posterior globus pallidus (p < 0.039). Recall from our post hoc analysis, only the ADHD patient group differed from the HCs (Bonferroni-corrected p < 0.001). We also observed significant interactions between the Chapman physical anhedonia and pairwise ROI functional relationships in the left dorsal anterior putamen and left crus I (p = 0.048), as well as between the left dorsal anterior-medial thalamus and right dorsal posterior putamen (p = 0.005). Both of these ROI pairs were significant according to our post hoc analysis for the SCHZ patient group (Bonferroni-corrected p < 0.001). Finally, we observed significant interactions between Chapman social anhedonia and the bipolar II risk scale score and between the left ventral anterior inferior anterior thalamus and the left DMN medial prefrontal cortex (p < 0.021). Our post hoc analysis revealed that only the SCHZ patient group differed from the HCs (Bonferroni-corrected p = 0.013).

#### 3.3.2. Task conditions (290)

In the PEncode condition, we observed significant interactions between three regional pairs across the impulsivity subscale, social anhedonia subscale and bipolar II risk scale scores. In the first pairwise ROI set (right ventral anterior thalamus with the left posterior putamen), we observed interactions with the Dickman impulsivity score (DIS) functional total and positive subscales, as well as the bipolar II risk scale (p < 0.042). Post hoc analysis of multiple comparisons revealed the ADHD and SCHZ patient groups differed from the HCs (Bonferroni-corrected p < 0.004). In the second pairwise ROI set (right caudate body with the right ventral posterior lateral thalamus), we observed interactions with the DIS dysfunctional total and negative subscales (p < 0.043). Post hoc analysis revealed the ADHD patient group differed from the HCs (Bonferroni-corrected p = 0.002). Lastly in the third pairwise ROI set (left ventral anterior thalamus with the left hippocampal head), we observed interactions with the Chapman social anhedonia subscale (p = 0.041). Again, post hoc analysis revealed the SCHZ patient group differed from the HCs (Bonferroni-corrected p = 0.039).

We observed interactions in both PAM retrieval conditions, between one regional pair in each condition with assessments. In the PCorrect condition, cerebellar crus I with the right crus II showed interactions with DIS functional total and positive subscales, as did the bipolar II scale (p < 0.026). Post hoc analysis revealed the ADHD patient group differed from the HC group for this ROI set (Bonferroni-corrected p = 0.025). In the PIncorrect condition, the left hippocampal head with the left dorsal anterior medial thalamus exhibited an interaction with the ASRS (p = 0.045). Post hoc analysis revealed the ADHD patient group differed from the HC group for this ROI set (Bonferroni-corrected p = 0.035).

## 4. Discussion

Emerging evidence across various psychiatric disorders, abnormal functional integration of CTC circuitry is associated with impaired task-related coactivation of brain regions (Alves et al., 2019; Anticevic et al., 2014; Ferri et al., 2018; Hwang et al., 2022; Matsuo et al., 2013). Using FNC approaches we address three areas overlooked in psychiatric neuroimaging research: CTC integration characteristics across various psychiatric disorders, state-dependent cognitive adaptation, and relation to clinical assessments. Our analyses revealed state-dependent aberrant relationships engaging specific regions of the CTC circuit across SCHZ, BIPOL, and ADHD patients compared to HCs. Furthermore, our study demonstrated, in both task and task-free conditions, regional connectivity within the CTC circuitry exhibit interactions with symptom and trait assessments congruent with psychiatric diagnoses. These findings support the notion of cognitive control imbalances relate to severity of behavioral symptoms, indicating shared relevance for state-dependent cognitive processes.

We first establish CTC circuitry brain regions were impacted across all patient groups relative to healthy participants at rest. From this established baseline, task conditions reflect regional adaptive cognitive control during declarative memory encoding and retrieval. Consistent to findings predominantly in the SCHZ patient group, we showed significant hypo- and hyperconnected regions associated with the CTC circuitry at rest (Fig. 1). For example, hyperconnected medial dorsal thalamus with the basal ganglia (Hwang et al., 2022; Matsuo et al., 2013) and hypoconnected to cortical areas such as the medial prefrontal cortex (Anticevic et al., 2014; Ferri et al., 2018; Scangos et al., 2023). Further, we observed hypoconnected hippocampus with the medial prefrontal cortex. However, hyperconnected ventral anterior thalamus with the medial prefrontal cortex and yet hypoconnected with the cerebellar crus II were also present (Habas, 2021; Schmahmann et al., 2019; Stoodley et al., 2016; Stoodley & Schmahmann, 2018). Reports suggest broad hyperconnected cerebello-thalamo-cortical circuitry is robust and may be a state-independent hallmark of psychosis in a task-free context (Hwang et al., 2022). Our evidence suggests regional hyper and hypoconnected integration.

Interestingly, declarative memory conditions revealed a strong differential regional response, more predominant in the ADHD patient group (Fig. 2). Given the task condition, regional patterns reflect functional engagement of the anterior insula, subcortical, and cerebellar regions. Task conditions involved different regional integrations than at rest but, at some level, involved the ventral and dorsal anterior thalamus during the declarative encoding and retrieval conditions. These findings suggested that the connections were intact at rest, but with an opposing balance between the brain and cognitive reserve to cope with brain pathology (Medaglia et al., 2017).

We chose symptom and trait assessments ascribed to primary diagnoses but sensitive to declarative encoding and retrieval memory tasks (Table 1). For example, where a more predominant effect was present in the SCHZ group than in the ADHD group for a given region in both task and task-free conditions, there is an interaction with Chapman social anhedonia. However, why similar regional integrations according to patient group may or may not interact with the same assessment is unclear. For example, in the task-free condition, cluster 4 had three integrated connections from the left dorsal anterior thalamus targeting the bilateral dorsal posterior putamen and left ventral posterior putamen, and with similar effect sizes according to treatment group for each paired-region (Fig. 1). However, only the integration of the left dorsal anterior thalamus with the right dorsal posterior putamen was associated with the Chapman physical anhedonia subscale (Table 2). A trending set of interactions did not include the Chapman physical anhedonia subscale for the integration between the left dorsal anterior thalamus and the left dorsal posterior putamen (Supplemental Table 1). This is consistent with purported links between tasks with varying demands during the coordination and evaluation of task performance (Eckert et al., 2009) and implicated in patients with psychosis and anxiety (DeSerisy et al., 2020; Jimenez et al., 2016; Rubia, 2018; Vieira et al., 2021; Wen et al., 2021). Taken together, these observations suggest that dysfunctional integration of regional CTC circuitry is more sensitive in discriminating particularly between schizophrenia and ADHD, than is clinical assessment alone. Given the effect size in our FNC analysis, it is also possible that declarative memory tasks may not be appropriate for detecting functional changes to distinguish bipolar II patients from schizophrenic patients and ADHD patients in this paradigm. However, an interaction existed between the trait risk scale for bipolar II with the right (not the left) ventral anterior thalamus during the encoding task. As expected, task and task-free conditions in concert with clinical assessments provide a better functional context for understanding the nature of the underlying etiology across the patient groups.

## 5. Conclusion

In conclusion, our study establishes that brain regions of the CTC circuit exhibit state-dependent patterns in patients with SCHZ, BIPOL, and ADHD. Additionally, these patterns interact with clinical symptoms and trait assessments, suggesting cognitive control imbalances, potentially mediated by thalamic regions, particularly the ventral anterior complex. These observations are consistent with the reported contrast between task-related and resting wakefulness resulting from deactivation of brain regions in the DMN. Data supports DMN integration of the basal forebrain, cerebellar subregions, striatum, and the thalamus, where altered connectivity was observed in schizophrenia and ADHD (Alves et al., 2019). Structurally, connectivity emanate from the ventral anterior thalamus to the basal forebrain, cerebellar regions, and mesial temporal regions (Alves et al., 2019; Palesi et al., 2015). Functionally, increased DMN activation was observed during episodic and semantic memory (Alves et al., 2019). Conversely, DMN regions show decreased activity during attention-demanding tasks (Alves et al., 2019). It is plausible that dysfunctional regional integration of the CTC circuit, particularly via the ventral anterior thalamus, is associated with functional imbalances in the DMN.

Our findings in this context further strengthen the transdiagnostic potential of cognitive control imbalances mediated by specific thalamic regions and nonmotor regions of the cerebellum. Taken together, this evidence contributes to the understanding of cognitive dysmetria as a shared continuum across multiple psychiatric disorders.

## Figures and Tables

**Table 1:** The demographic and clinical data for the participants.

		HC	SZ	BIPOL	ADHD	Statistics	
		Mean ± SD	Mean ± SD	Mean ± SD	Mean ± SD	F-Stat	* p value
Sample Size (female/male)		39 (16/23)	27 (6/21)	38 (17/21)	31 (14/17)		
Age (years)		32.7 ± 9.3	36.4 ± 9.3	34.7 ± 8.8	31.1 ± 9.4		
* Post-Hoc Games-Howell Multiple Comparisons (assume unequal variances, p < 005 significance between HC and Diagnosis)							
**Symptoms**							
Adult Self-Report Scale v1.1 Screener (ASRS)		9.3 ± 2.6	10.4 ± 4.3	13.1 ± 5.2*	15.8 ± 3.9*	16.88	< 0.001
Hopkins Symptom Checklist (HSCL)	Interpersonal Sensitivity	0.4 ± 0.4	1.1 ± 0.7*	1.1 ± 0.8*	0.7 ± 0.5	11.44	< 0.001
	Somatization	0.2 ± 0.2	0.7 ± 0.5*	0.7 ± 0.7*	0.3 ± 0.2	10	< 0.001
	Anxiety	0.3 ± 0.3	0.9 ± 0.7*	0.7 ± 0.7*	0.4 ± 0.4	9.94	< 0.001
	Obsessive-Compulsive	0.5 ± 0.4	1.2 ± 0.6*	1.2 ± 0.8*	1.2 ± 0.8*	10.32	< 0.001
	Depression	0.4 ± 0.4	0.9 ± 0.7*	1.0 ± 0.6*	0.6 ± 0.4	7.76	< 0.001
	Global Severity	0.4 ± 0.3	0.9 ± 0.5*	0.9 ± 0.6*	0.6 ± 0.4*	12.52	< 0.001
**Traits**							
Dickman Functional and Dysfunctional Impulsivity Scale	Functional Total	6.5 ± 2.6	5.5 ± 2.5	5.8 ± 3.0	6.6 ± 3.0	1.08	0.36
	Functional Positive	3.2 ± 1.8	3.2 ± 1.8	3.3 ± 1.8	3.4 ± 3.0	0.14	0.94
	Functional Negative	3.3 ± 1.4	2.3 ± 1.3*	2.5 ±1.5	3.2 ± 1.7	3.94	0.01
	Dysfunctional Total	1.3 ± 1.8	4.5 ± 3.1*	6.2 ± 4.3*	4.9 ± 2.9*	16.59	< 0.001
	Dysfunctional Positive	0.8 ± 1.8	3.4 ± 5.2*	4.3 ± 3.0*	3.6 ± 2.3*	15.03	< 0.001
	Dysfunctional Negative	0.46 ± 0.6	1.1 ± 1.4	1.9 ± 1.5*	1.3 ± 1.2*	11.52	< 0.001
Chapman Scales	Physical Anhedonia	10.4 ± 6.3	16.5 ± 7.2*	16.6 ± 9.1*	13.6 ± 6.0	5.83	< 0.001
	Social Anhedonia	9.4 ± 6.2	16.0 ± 6.2*	16.0 ± 7.1*	14.1 ± 7.6*	7.57	< 0.001
Scale for Traits that Increase Risk for Bipolar II Disorder		10.7 ± 4.8	15.4 ± 5.6*	17.5 ± 6.2*	14.6 ± 3.6*	11.52	< 0.001

**Abbreviations**: HC: Healthy Controls; SZ: Schizophrenia; BIPOL: Bipolar Disorder; ADHD: Attention Deficit Hyperactivity Disorder; F-stat: F-statistic from comparing multiple independent variables

**Table 2:** Between-subject effects testing interactions between significant pairwise ROIs (covariates) and symptom/trait assessments (factors).

	ASRS	HSCL-OC	HSCL-Dep	HSCL-GS	DIS-FuncTot	DIS-FuncPos	DIS-DysFuncTot	DIS-DysFuncNeg	Chap-PhysAnh	Chap-SocAnh	BipolarII Scale
	*Full-Factorial (task-free) and Factor-by-Covariate (task) Generalized Linear Model MANOVA Tests of Between-Subjects Effects where previously determined ROIs from survived FNC clusters covary: F-Statistic (p value) shown for p < 0.05 significance. AH other p values for p < 0.10.* *Post-Hoc Bonferroni multiple comparisons (assume unequal variances, p < 0.05 significance between HC and Diagnosis).*										
**Task-free (of 231 FNC Clusters)**											
**Cluster 2**: L-DA-PUT w/L-Crus I									3.97 (p = 0.048)		
**Cluster 4**: L-THA-DAm w/R-PUT-DP									8.32 (p = 0.005)		
**Cluster 15**: L-CAU-tail w/R-pGP		4.37 (p = 0.039)	7.78 (p = 0.006)	5.09 (p = 0.026)							
**Cluster 35**: L-THA-VAia w/L-DefaultA-PFCm										7.49 (p = 0.007)	5.45 (p = 0.021)
**Encoding Task (of 185 FNC Clusters)**											
**Cluster 2**: R-CAU-body w/R-THA-VPl							4.19 (p = 0.043)	6.14 (p = 0.015)			
**Cluster 2**: L-THA-VAia w/L-HIP-head										4.26 (p = 0.041)	
**Cluster 9**: R-THA-VAip w/L-PUT-DP					4.22 (p = 0.042)	9.75 (p = 0.002)					5.97 (p = 0.016)
**Retention Task - Congruent (of 166 FNC Clusters)**											
**Cluster 1**: R-VIIb - Crus II w/R-Crus II					5.06 (p = 0.026)	6.39 (p = 0.013)			5.14 (p = 0.025)		
**Retention Task - Incongruent (of 204 FNC Clusters)**											
**Cluster 7**: L-HIP-head w/L-THA-DAm	4.11 (p = 0.045)										

**Abbreviations**: HC: Healthy Controls; SZ: Schizophrenia; BIPOL: Bipolar Disorder; ADHD: Attention Deficit Hyperactivity Disorder; ASRS: Adult Self-Report Scale v1.1; HSCL: Hopkins Symptom Checklist; DIS: Dickman functional and dysfunctional impulsivity scale; Chap: Chapman Scales for social anhedonia and physical anhedonia; L: Left; R: Right; DA: dorsal anterior; PUT: putamen; Crus: cerebellar crus; THA: thalamus; DAm: dorsal anterior medial; DP: dorsal posterior; CAU: caudate; pGP: posterior globus pallidus; VAia: ventral anterior inferior anterior; PFCm: medial prefrontal cortex; HIP: hippocampus; VIIb: cerebellar lobule VIIb; df: degrees of freedom; F-stat: F-statistic from comparing multiple independent variables

## Highlights

- Corticothalamocerebellar communication has been implicated in cognitive dysmetria.
- During task and task-free conditions, are cortico-thalamo-cerebellar regions similarly engaged across all three psychiatric disorders?
- Functional network connectivity (FNC) clusters of the cortico-thalamo-cerebellar circuitry demonstrate that there is a functional integration imbalance in both task and task-free conditions compared to healthy subjects, which differs across all three psychiatric disorders.
- These FNC findings are congruent with some clinical assessments but provide broader regional insights than assessments alone.
